# A study of the mechanism for intergenerational transmission of gender roles in single-parent families

**DOI:** 10.1016/j.heliyon.2023.e22952

**Published:** 2023-11-28

**Authors:** I-Jun Chen, Zisong Chen

**Affiliations:** a School of Education, Soochow University, Dushuhu Campus, No. 1, Wenjing Road, Suzhou Industrial Park (SIP), Suzhou, 215123, China

**Keywords:** Gender roles, Grounded theory, Intergenerational transmission, Single-parent family, Transmission mechanism

## Abstract

The divorce rate in China is rising yearly, and the concept of marriage is changing, triggering many social topics related to single parents. Among them, gender-awareness education for children in single-parent families is particularly worthy of attention, as there has been insufficient exploration of how parents transfer their gender role concepts to their children. This study conducted in-depth interviews with 58 single parents and children from 29 families in Suzhou and constructed the mechanism for intergenerational transmission of gender roles in single-parent families based on grounded theory. It found that single parents' gender stereotypes, the starting point of the intergenerational transmission mechanism, have been diluted. It affords them a more enlightened attitude towards child-rearing style, and they expect more equality in the gender role of their offspring. However, in some parents' actual parenting process, when the children's sexualization behaviours exceed their acceptance range, they will communicate with children in an authoritative and didactic way to “correct” the children's behaviour. In addition to direct verbal instruction, parents' expectations of their children's gender roles will be implicitly conveyed through various parent-child interactions in daily life to affect the formation of their children's gender roles. In the intergenerational transmission of gender roles, external people (such as grandparents, teachers and peer groups) have horizontal socialisation effects on children to modify or strengthen the results of gender education from their parents. Under the comprehensive influence of the above factors, the children's gender roles are finally determined. These studies expand previous theories and studies and have significant theoretical and practical implications.

## 1 Introduction

With the development of the times, the divorce rate is increasing year by year in the world. China is a developing country, and the concept of marriage among Chinese families is also changing. In 2020, there will be 4.3 million couples divorcing in China, and the divorce rate will reach 3.1 ‰. The rising divorce rate has brought about many global social problems. For example, the economic and social resources of single-parent families may be relatively insufficient [1,2], which leads to the poverty of single-parent children [3,4], and more likely to drop out of school [[5], [6], [7]], unemployment, unmarried pregnancy and other phenomena [[8], [9], [10]]. Changes in family structure may also adversely affect single-parent children's physical and mental health [11], such as being trapped in the pain of abandonment and the illusion of parental reunification [10,12]. In addition, the absence of a particular parental gender role makes the gender-awareness education of single-parent children a particular concern, as it informs their concepts of marriage [13], values, and social adjustment [14]. If the gender role development of the offspring is not effectively guided, it may lead to their “impression bias” towards gender, i.e., a preference for the female or male [15], and being insecure about the future intimate relationship [16,17] Previous studies on single-parent children's gender role development have mainly been quantitative, exploring the relationship between gender roles and other variables, such as self-compassion [18] and life satisfaction [19]. Or comparing the differences in gender role development between single-parent and two-parent children [20]. There is less exploration of how parental perceptions of gender roles are transmitted to their children. Qualitative research methods are uniquely positioned to provide insight into social phenomena through rich description and analysis [21]. It allows for an unbiased exploration of family-level microsystems and different information about parent-child interactions. This approach reveals the complex intergenerational transmission of gender roles in single-parent families, allowing researchers to discover new theoretical perspectives that will enhance understanding and support future research and practice.

The gender role is one of the important contents of individual socialisation, which is the gender and personality characteristics formed through a long process in a specific culture [22]. It refers to a set of norms of behaviour corresponding to gender consciousness in socialisation through imitation learning [23]. The research on gender roles has evolved over a long period. Studies before 1970 were predominantly unidimensional and considered male and female sexual characteristics as two extremes of a continuum. However, many scholars have questioned this polarised view of gender roles. For example, the psychoanalytic school's concept of “the subconscious dualisation” states that there is no pure male or pure female for human beings, in neither the psychological nor biological sense. It states that humans are born with both genders' biological and psychological characteristics, including the feminine subconscious (Anima) and the masculine subconscious (Animus). Further, Bem divided gender roles into four types: androgyny, masculinity, femininity, and undifferentiated [24], and developed the Bem Sex Role Inventory (BSRI) [25]. It is now generally accepted that androgyny is more popular in modern society.

The family microsystem has the most direct, initial, and powerful influences on cultivating children's gender roles [26]. In raising children, parents will pass on the characteristics of different genders to their children, including ideologies, behaviours and social norms, even decision-making, which form the phenomenon of intergenerational transmission [[27], [28], [29], [30]]. Intergenerational transmission is the phenomenon whereby parents' abilities, ideas, behaviours, and social status are passed on to their children [31]. This is consistent with Social Learning Theory, which holds that children acquire their gender roles primarily through direct education and observational learning [32]. However, scholars have different views on the influence of the absence of one parent on the intergenerational transmission of gender roles.

Most studies have found that single parenthood negatively impacts children's gender roles. The children may have difficulty acquiring opportunities to learn the behaviour corresponding to their physiological sex if the single parents have a different physiological sex than their children. This discourages their formation of androgyny and affects their identification with a certain gender role [33]. Some researchers believe that the healthy development of individual gender roles is carried out in a gender balance structure [34,35]. But the structure of gender balance within single-parent families has changed the triangular relationship (between father, mother, and child) into a linear one [36] The multiple roles of male and female (gender) and older and younger (seniority) have been reduced to just father/mother and son/daughter (generation). The complex family structure became relatively simple, which is not conducive to children's exposure to diverse gender role behaviours. At the same time, single parents, due to limited energy, may pay more attention to satisfying their children's material needs than the development of gender roles [20].

However, it has been argued that the gender roles of parents and children may be more flexible in single-parent families [37]. Research has shown that single parents have more androgynous gender role attitudes toward their children, and daughters have more flexible gender roles in single-mother families [38]. A study in China found that the proportion of high school students with undifferentiated gender roles in single-parent families was significantly lower than that in two-parent families, and there was no difference in the proportion of androgynous gender role duality between the two types of families [39] Suggesting that changes in family structure do not necessarily have negative effects on the intergenerational transmission of gender roles. Although the mechanisms underlying this phenomenon have not been fully studied in the context of China's patriarchal social structure, parents usually educate boys strictly about their gender roles, while girls are relatively relaxed [40]. Thus, females who exhibit masculine characteristics are more likely to be publicly accepted than males who exhibit feminine characteristics. Single parents have to take on the dual tasks of working outside the home and caring for the family. Therefore, single fathers may be gentle, tolerant, and caring, and single mothers may be bold, independent, assertive, and so on. Children will be less likely to perceive these androgynous traits of single parents as a manifestation of gender role deviation but as socially acceptable qualities. So, they may be more likely to accept the androgynous gender roles [37,38,41,42]. In addition, Chinese families usually attach great importance to the concept of “extended family”, and grandparental education is widespread in China. Therefore, grandparents play an important compensatory role in supporting grandchildren's upbringing in divorced families [43,44]. Although single parents were still primarily responsible for their children's upbringing, grandparents were involved in household tasks such as cooking and laundry [[45], [46], [47]]. A social study conducted in 30 Chinese provinces showed that nearly 60 % of the elderly assisted in their grandchildren's upbringing. This suggests that single-parent families in China are not the same as “single-sex families”. Single-parent children can also acquire gender concepts from grandparents and relatives. This phenomenon increases the particularity of intergenerational transmission of gender roles in modern single-parent families in China.

In conclusion, the family structure changes due to parental divorce, and the intergenerational transmission of gender roles may also be affected positively or negatively. It is worth further exploring how the gender role constructed by parents transfers to children in single-parent families in a single-parent family system.

## 2 Method

### 2.1 Study area

Suzhou, Jiangsu Province, which is characterized by “rapid economic development”, “significant divorce rate” and “rapid integration of migrant population”, occupies an important position in China's economic and social development. In 2022, the Gross Economic Product (GEP) of Suzhou is $33.381 billion, ranked as sixth in China [48]. Suzhou City had a resident population of 12.911 million, with a net inflow of 41.6 %, ranking third in the China [48]. Population mobility is often affected by geographical, economic and social aspects. Families with floating populations usually face the dilemma of family members being scattered between two or more places. And they have been separated from their spouses, children and other family members for a long time, which increases the risk of divorce. Although Suzhou's GEP ranks sixth among Chinese cities and the inflow of population ranks third, its divorce rate in 2022 is as high as 38.12 %, which is not only higher than the Chinese average of 7.39 % [49], but also higher than Shanghai, Guangzhou, Shenzhen and other first-tier cities in China. However, the number of single-parent families is bound to increase significantly with the number of divorces. Therefore, based on the characteristics of economic development and social structure, it is worth further research on the development process of the gender role of single-parent families in Suzhou.

### 2.2 Participants

This study adopted an intensity sampling strategy based on theoretical sampling principles to select typical cases with high information intensity. The only criterion for sample selection was whether it could provide new insights to build or deepen the theory [50]. We did not specify the number of interviews at the beginning of the study but based it on data saturation. The ultimate goal of the interviews was to ensure stable consistency between categories, variation and saturation of theoretical implications, and depth of focus.

The researchers, with the help of Suzhou's local government, contacted 13 middle schools; data saturation was reached after in-depth interviews with 58 people from 29 single-parent families (offspring and their single parents). A single-parent family refers to a family in which only one parent lives with the child due to widowhood, divorce, separation, or being unmarried [51]. In order to avoid subjective bias and error and to ensure the reliability and validity of the study, we arranged two different groups of researchers to conduct separate in-depth interviews with parents and children. Through this way, we could analyze the data from different perspectives and conduct cross-validation to obtain mutually corroborating information which could provide more comprehensive and accurate data, thus making the interview study more scientific and reasonable. The children in these families were all in middle school, with a grade distribution from junior to senior high school (grades 7–11). The interviewees were divided into four categories based on family structure: single mother-daughter families (nine), single mother-son families (nine), single father-daughter families (eight), and single father-son families (three). Informed consent was obtained from all 29 guardians and their children (See Table 1).

**Table 1:** Demographic background of respondents.

	Respondents (N = 29 families)
Offspring School situation	
Junior high School	20
Senior high School	9
Offspring Grade	
Average	≈8
Range	7∼11
Offspring gender	
Male	12
Female	17
Parental gender	
Male	11
Female	18
Family structure	
Single mother-daughter families	9
Single mother-son families	9
Single father-daughter families	8
Single father-son families	3

### 2.3 Data collection and analysis

At the beginning of the study, the researchers contacted 13 high schools with the help of the local Suzhou Education Bureau and obtained consent from the school authorities. Informed consent was obtained from all 29 guardians and their children for this study. Approval was also obtained from the ethics committee of Soochow University. The researchers explained the purpose and procedures of the interviews and that participation was completely voluntary and could be terminated at any time. The interviews were carried out over a span of 40 days, commencing in mid-November 2020 and concluding at the end of early January 2021. All participating parents were divorced and had been raising their children alone for a stable period. Researchers assigned each participant a number to ensure anonymity. The number is made up of four parts: (1) the first letter of the parent's name, (2) the first letter of the child's name, (3) a number indicating the order in which the interview was conducted, and (4) a lowercase letter indicating the specific member of the family structure. Thus, FS1 (f) represents the father in father-son family number 1 and MD2 (d) represents the daughter in mother-daughter family number 2.

This study used semi-structured interviews. The researchers conducted pre-interviews with families around us before the in-depth interviews. The focus was on the issue of “gender role education in single-parent families” to provide a basis for the formal interview outline. All interviews were conducted on campus to ensure that the children were familiar and comfortable with the environment. And all were conducted in Chinese to avoid language ambiguity or barriers. The interviewers were psychology researchers trained in communicating with interviewees to ensure their ability to complete the interviews. The participants' demographic information was surveyed and recorded during the formal interviews. Questions related to single parents’ gender roles were emphasised, such as “What kind of personality do you think you have? What kind of career do you want your child to pursue in the future? What factors do you consider when taking care of your children in your daily life?“. There was no time limit for the interviews; it ended when the information reached saturation and the interviewees had nothing more to add. All interviews lasted between 40 and 80 min and were recorded throughout. The researchers converted all recordings into verbatim transcripts for subsequent analysis within 24 h of the interviews, yielding over 500,000 transcribed words.

This study adopted a qualitative research strategy guided by Glaser and Strauss's Grounded Theory [52]. Its' goals were to expand the existing theoretical framework, localize it after scientific analysis of the textual materials, and construct a discourse system aligned with the contemporary Chinese context. To ensure the theory's final development, this study adopted a cyclical research model for textual analysis, consisting of three levels: open coding, axis coding, and selective coding. Using QSR NVivo 11.0 qualitative analysis software, we analyzed the textual interview data sentence by sentence, with “intergenerational gender role transmission” as the core issue. The recurring meaning units in the case data were searched for, and then the original textual data based on different meaning units to form different nodes were split and merged. The nodes were further classified and the initial codes were constructed as the base units for subsequent analysis [53]. Thus, realizing the theoretical coding and construction of non-numerical, unstructured textual materials. The final theoretical results were constructed bottom-up on top of the three-level coding.

## 3 Results

### 3.1 Parental gender role perspectives

Parents’ gender role concepts mainly include recognising their gender role characteristics and having views on the different gender concepts in social culture.

This study found that single parents' self-awareness was consistent with their physiological sex for most of their gender role characteristics. However, they also often had androgynous gender role traits. Single parents have to take on the responsibilities of both gender roles at work and in life, which may magnify their heterosexuality. Single parents who split up have to assume both gender roles at work and in life, which may amplify their androgynous gender role traits. This trait was evident in single mothers; as MS1(m) stated, ‘*I used to think I could only be someone who did simple work, who couldn't do anything. But when I got divorced, I felt I could do anything and didn't need to prove it to anyone*’. In addition, children in single-parent families had distinct feelings about their parents' gender behaviours that differed from their physiological sex and believed their parents played a more complex role in the family. As FD8(d) said, ‘*My dad ties my hair every morning … and I think, well, he's just like a dad and a mom at the same time*’. Such interactions may influence children's perceptions of their gender roles. As FD8(d) mentioned in the follow-up interview, ‘*I am considered a tomboy all through the school; that's just who I am*’.

Single parents were less likely to agree with division of labour and gender differences, such as ‘*the man dominates the outside, and the woman dominates the inside*’, perhaps because they often need to work outside the home and take care of the family. They believed the distinction between duties outside and inside the home should not just be based on physiological sex, but on ‘*whoever has the time to do it*’ (MS2(m)), or ‘*Whoever has the ability to earn money. It is not necessary to divide who is the main worker*’ (MS1(m)). They also believed that the differences between men and women were mainly physiologic (such as physical strength), so they held more open views on those occupations with obvious gender labels. For example, MS2 (m) said, ‘*I think it's great*’ that men choose to be kindergarten teachers or nurses as a career. These perceptions of single parents implicitly influenced their offspring's perceptions of gender roles and the division of labour in the family. For example, the daughter in the FD4 family, whose father was responsible for household chores, disagreed with the traditional division of labour between males and females, saying, ‘*I grew up in an environment where it was unnecessary for men outside and women inside, like my father can do (housework) and he does it when he has time, so I think men should do housework too*’. However, this study also found that a few parents still had traditional gender stereotyping. For example, a minority of the single parents interviewed believed that women were more attentive than men and, therefore, better suited to domestic work; men, on the other hand, were better suited for heavy labour or mechanical jobs. Overall, only a small number of respondents held this view. The single parents interviewed were more inclined to hold gender equality views.

### 3.2 Parental awareness of gender-role parenting

Parents’ gender-role parenting of their offspring can be divided into two aspects: parent-led gender-role expectations for their offspring and child-led gender-role-free development.

Some interviewed parents believed that their own families could not provide a strong guarantee for their children after a divorce. Therefore, single parents emphasised their offspring's “independence”, “strength”, and “responsibility” when it came to gender role expectations. For example, FD7 (f) mentioned the requirements for her daughter, ‘*You can't depend on anyone because this society is very realistic, and if you depend on others, you will be eliminated by society*’. Single mothers have similar parenting attitudes towards their daughters. For example, MD1's (m) requirement was that her daughter be ‘*an independent person who can help others and also have a sense of responsibility*’ and MS1(m) emphasised ‘*educating him to be a man with responsibility and commitment*’ when dealing with her son. Single fathers' requests to their sons were more direct. FS2 (f), for example, told his son, ‘*You have to be responsible*’, while FS3 (f) emphasised the importance of strength, telling his son he ‘*must be strong because you are a boy*’.

Single parents emphasised the ‘*stability of occupation*’ regarding what they wanted their sons or daughters to do in the future. MS9 (m) expected her son to have ‘*a stable job with a good skill*’, MS6(m) hoped her son would work as an ‘*office clerk*’, and FD1(f) hoped that he could help her daughter ‘*find a stable job’*, showing that single parents did not show gender-specific expectations regarding their children's careers.

Most parents believed they gave their offspring freedom during their gender role development, respecting their independent choices. This was mainly reflected in parents giving their adolescent children some right to decide their affairs. For example, most parents allowed their children to choose clothes and interest classes independently and participate in some outside-the-home activities. Some parents, such as MD2(m), even participated in those activities with their children: ‘*Because I have more time, I also cooperate with her. For example, we wear a Chinese costume (traditional Chinese dress), and go for a walk on Pingjiang Road (a historical street in Suzhou). I feel quite good*’. Parents also expressed respect for their children's future job aspirations; for example. MS5(m) said, ‘*Since he chose this, like a male kindergarten teacher or a gynecologist, right? I'm sure I'll* support *him*’.

However, further research showed that although single parents allowed their offspring to develop their cognition freely, it was still difficult for them to implement their behaviours fully. The parents praised their offspring and reinforced behaviours that conformed to their expectations, but criticized behaviours they saw as unacceptable. For example, FD8(f) believed his daughter should not use lipstick in junior high school, so when she unintentionally took out her lipstick, he said angrily, ‘*I'll give you a chance today; I won't say anything to you, do you know how to do it? Throw it in the trash*’. After that, he stopped giving his daughter pocket money and repeatedly said, ‘*I'm very angry, where did you get the money, how can you buy lipstick, how old are you still*’.

### 3.3 Intergenerational transmission paths

The parents transmitted their gender role concepts and awareness to their offspring through explicit (direct praise or criticism) and implicit (various other forms of parent-child interaction) means. There were also gender differences in the proactivity of single parents’ parent-child interactions.

Single parents directly informed the children through verbal instruction and indirectly conveyed their expectations through diverse parent-child interactions. For example, in most single-parent families, children were required to share the household chores to compensate for the absence of one parent. In particular, the children in the “Father-Son” families were often required to do housework such as ‘*wash the dishes after dinner* (FS2(s))’. In addition, tutoring was a special and very common form of parent-child interaction. The entrance exam is one of the most important educational events in a Chinese family; as such, Chinese parents attach great importance to their offspring's academic performance, and the children's test scores generally inspire praise or criticism. Different gender role characteristics were reflected in the tutoring process, impacting the children's gender role development. For example, MD8(d) mentioned that her parents had different tutoring styles: ‘*Dad was there to teach me, and then I still didn't know how to do it, so he hit me, it made me cry. Then my mom scolded him, and then my mom taught me. The good kind of guidance is a very gentle kind of teaching me, and I did not cry, just listen to her guidance*’. However, in single-parent families, the offspring relied on the sole parent's teaching and had to “correct” their own “mistakes”. As MS9(s) mentioned, ‘*when there are most arguments about tutoring, it's always my mom who is right and good, and I always wrong*’.

Several parents also said they had little opportunity for parent-child interaction because they were busy with work. FD5 (f) had little time to communicate with his daughter because of his early morning and late evening work: ‘*I work late and come back after she has gone to bed, and then I leave very early in the morning. Basically, there is little opportunity for communication*’. FD6(d) said, when asked about her father's companionship, ‘*he is at work and has no time for me*’. The parents were aware of this problem but did not have good ways to deal with it. For example, MS9 (m) mentioned, ‘*Now I do not have much time to take care of him or to tutor him, which may cause him not develop some good habit*’.

Single mothers showed a more positive and proactive willingness to communicate with their children than single fathers. Most single mothers in the interview were more inclined to express their love to children directly, saying that they were willing and able to ‘*talk like friends*’ with their children. MD2(m) said, ‘*I know she loves me too, but I want her to express something. Be able to express it, meaning not just let me feel it, let me hear or see it, express it directly*’. Such expressions were less common among single fathers, who were usually more restrained in dealing with their children and showed passivity in parent-child communication. For instance, when FD6(f) mentioned parent-child communication, he emphasised, ‘*I am sometimes embarrassed to say it, it is my mother who talks to her*’. FD5(f) also showed passive characteristics in parent-child communication; when communicating with his daughter, he usually ‘*just made a quick comment*’, adding that ‘*it's up to her to listen or not*’. The offspring's willingness to communicate with their parents will be weakened if the single father is passive, retreats in parent-child communications, and is unwilling to accept their gender role teaching. As FD3(d) mentioned in the interview, ‘*Usually when he says these things like how girls should be, I just change the subject or shut up immediately*’.

### 3.4 Impact of single-parent family structure on gender education

There are many special parenting challenges in single-parent families; divorce can lead directly to personality changes in both parents and children, and gender role transmission is more difficult when the child's physiological sex differs from their single parent's. Single parents must consider how to communicate with their offspring about divorce.

Parental divorce is an important experience for whole-family life. The interviews revealed that single parents' views on the division of labour and their perceptions of their gender role characteristics changed after their divorce as they had to bear a burden once shared by two people. Some parents thought themselves fully capable of coping alone: ‘*After the divorce, I think I can do everything*’ (MS1(m)). However, some single mothers expressed their helplessness and feelings about the absence of men in the family. MD9(m), for example, mentioned that ‘*Women, without men, definitely can't do anything. Have no motivation to do anything*’. In addition, single parents' androgynous characteristics were amplified after divorce; for example, MS9(m) mentioned that her personality became more masculine after divorce; ‘*So I feel like I've become a bit of masculinity and have to be strong myself*’. The offspring's gender role characteristics also changed in some respects; MS4(m) emphasised that her son became ‘*grumpy*’ and ‘*irritable*’ and believed ‘*the changes in the family that may have led to these negative changes. Before it was quite good*’.

The offspring's perceptions of a particular gender were also influenced by the single parent's post-divorce comments about their ex-spouse. When their comments were positive, their offspring were more likely to have a positive perception of the ex-spouse's gender; when they were negative, so were the offspring's perceptions. For instance, FS1(f) had a generally positive perception of his ex-spouse and they communicated about their child's education: ‘*I talk to his mother about the children's education, and what I don't understand about … I communicate with her occasionally about education issue*’. Accordingly, FS1 (s) had a more positive perception of female gender roles. Similarly, FD3 (f) had a negative evaluation of his ex-spouse, and his daughter (FD3 (d)) voiced disapproval of traditional female gender roles: ‘*Why must all girls be a great lady?*’

In addition, this study found that when the child had the same physiological sex as the ex-spouse, some parents confessed to projecting their perceptions of the ex-spouse onto their offspring, ‘*I might kind of like to look for his dad in him*’ (MS7 (m)). However, most offspring did not share this projection, which may be generalised as disapproval of certain gender roles. As FD3 (d) directly stated in the interview, ‘*My dad keeps saying how you're exactly like your mom … I wish dad wouldn't keep associating me with my mom like this way.*’ In contrast, such projections by single parents were not found when their offspring and ex-spouse's physiological sex differed.

In the study, most single parents felt guilty about the effect of their divorce on their offspring. FS1(f) said in the interview, ‘*Since our divorce, he misses his mother's love*’. FS3(f) also mentioned that his son missed his ex-wife after the divorce: ‘*he is separated from his mother and wants his mother very much. He misses his mother very much*’. They found it difficult to make up for this sense of loss and could do little more than ask their children to ‘*be strong*’ and ‘*not to cry*’. FS3(f) would say to her son, ‘*Be strong and don't cry out. Don't cry. But it is normal to cry, and I want to teach him that this is the way to be strong as a boy*’. All these interactions were integrated into their children's gender role development.

One of the main reasons single parents face difficulties in their gender parenting is that the dislocation of one parent's role results in the absence of a major role model from the child's gender role development process. Although the single parents thought there was no obvious difference between their and their ex-spouses’ work and life abilities (especially when their physiological sex differed from their child's), they still faced insurmountable difficulties in their gender role parenting. On the one hand, parents believed there was a lack of direct role models in their offspring's gender role development. For example, MS5(m) felt it difficult for single mothers to have the same ‘*power*’ as fathers when teaching their sons. She was concerned that her son ‘*might be girlish if he keeps living with me*’. On the other hand, single parents felt ‘*some things are not easy to talk about*’ (FD6(f)) when dealing with children of the opposite physiological sex, such as fathers dealing with daughters' menstruation. Single fathers clearly felt the parenting difficulties caused by the absence of one gender role from the family; FD5(f) kept saying: ‘*If only her mother were around, she could tell her about menstrual problems*’.

Single parents also had to spend more time working outside the home because the absence of one parent reduced the family's workforce, so they faced greater financial pressure. Thus, the single-parent children in the study often lacked their parents' companionship. The single parents felt helpless about this; as MS9(m) said, ‘*It's just that I spent less time with my children. Well, that I was probably too busy. I probably just, um, didn't have a good balance. I don't have a good balance, and I actually regret it a little bit now in terms of the children, but I had to survive first, right?*’ Children's gender role development was also somewhat affected. FD8(d) mentioned that she had to learn many things on her own because her single father had to work to secure the family's income; ‘*My father is the only one in our family who works and makes money, it was that time (parental divorce) when I couldn't even tie my own hair*’.

### 3.5 Influence of non-parents on children's gender role development

The external influences of others on children's gender role development in single-parent families came from two main sources: teachers and peer groups in the school, and the ex-spouse and family members other than the single parent.

The single parents interviewed indicated that their children spend more time in school than with them, suggesting teachers and peers could significantly influence their gender role development. Teachers' important influence in this research was shown mainly in their ability to reinforce or modify single-parent children's gender role upbringing. For example, FD7(d) stated, ‘*My father said I should be strong, and many teachers said the same thing*’. In addition, the children compared their behaviour with traditional social and cultural conceptions of gender roles, using their peers' behaviours as a reference object. For example, FD4(d) mentioned, ‘*I think the girls in our class are quite happy and lively, but I don't think they are like traditional girls. I can't find any more traditional girls in our class*'.

Single parents repeatedly mentioned grandparents' involvement in parenting, suggesting family members played a crucial role in children's gender role development, particularly when the single parent and child were of different physiological sexes. For example, in “Father and Daughter” families, grandmothers often had the responsibility of teaching granddaughters about biology and listening to their worries (traditionally considered a mother's duty); ‘*I used to tell my grandmother when I was upset, and she would always listen to me (FD5(d))*’.

In addition, the ex-spouse's influence over their offspring did not disappear after the divorce. The single parent's ex-spouse could give the children additional perspectives on gender roles, complement the single parent's gender-role parenting, and serve as a role model in the children's gender role development process. For instance, FD4(d) mentioned that ‘*my mother once said that girls already physiologically vulnerable, so you should be better in your career*’. The single parents were well aware of this influence. FD1(f), for example, believed his daughter's character was deeply influenced by his ex-wife: ‘*Her personality is the same as her mother's, which means that she has been quite sensible since she was a child*’.

## 4 Discussion and recommendations

**Fig. 1:**
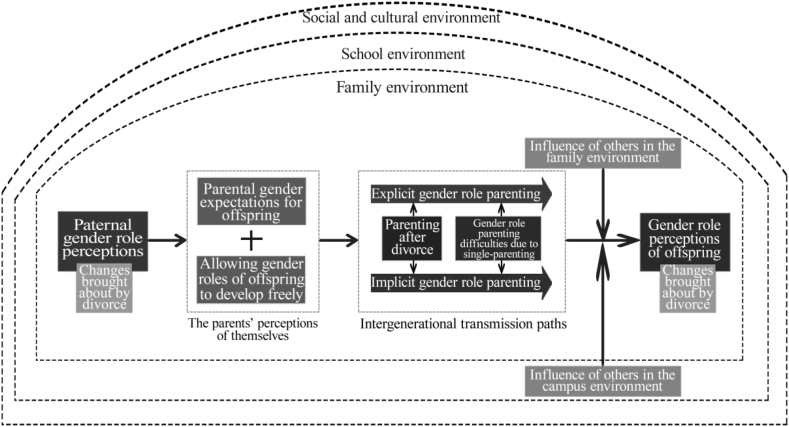
Intergenerational model of gender role transmission in single-parent families.

This study used qualitative research methodology to construct a mechanistic route for intergenerational gender role transmission in single-parent families (Fig. 1). It found that (1) single parents' gender perceptions were diminished compared to traditional stereotypes, perhaps due to post-divorce changes in family structure; (2) single parents' gender role perceptions were the starting point for intergenerational gender role transmission and influenced their gender role attitudes and behaviours. (3) single parents' gender-role parenting attitudes and behaviours were inconsistent with their cognition and practice; (4) the intergenerational transmission of gender roles in single-parent families followed two main pathways (explicit and implicit); and (5) in addition to their single parents, children's gender roles were also influenced by other family members (e.g., grandparents), teachers, and school peers.

### 4.1 Single-parent gender role perceptions were the threshold of intergenerational gender role transmission

Based on the interview data, this study identified single parents' gender role perceptions as the origin of the intergenerational gender role transmission mechanism in single-parent families, informing their gender parenting attitudes and behaviours and thereby influencing their children's gender role formation through both explicit and implicit transmission paths. The effects of changes in family structure were reflected in several aspects of the intergenerational transmission mechanism.

Single parents were more open to characteristics once thought gender specific; for example, being “considerate” was often considered a female characteristic. This result was consistent with previous studies [20,54]. They believed men and women could possess multiple characteristics and that individuals exhibited different personality traits independent of their physiological sex. On the one hand, this may be related to the increasingly refined division of labour in Chinese society [55], which has greatly increased job opportunities for women and made individuals more valued in the workplace, thereby encouraging single parents to have more equalised gender role perceptions [56]. On the other hand, it may be related to the fact that labour and responsibility are divided differently in single-parent families than in traditional two-parent families. Single parents have to take on financial and caregiving responsibilities, and there is an interplay between the family's division of labour and its concept of gender [57]. Therefore, single parents' dual role identity practices can be considered one of the important reasons for the dilution of gender stereotypes.

An individual's values can significantly predict their corresponding attitudes, influencing their decisions and behaviour [58]. Parents must behave with paternalistic altruism in their children's gender-role education, purposefully transmitting beliefs, preferences, and values to their children that they believe to be correct or beneficial [59]. The interviews in this study revealed that most parents emphasised personality characteristics like “independence,” “strength,” and “responsibility” in their gender role expectations for their offspring, while their expectations for children's future careers emphasised “stability,” regardless of gender. The single parents' gender stereotypes had faded in favor of more enlightened gender role parenting attitudes, making their gender role expectations for their offspring more egalitarian. This finding was consistent with previous studies [60,61]. Single parents' parenting behaviours were influenced significantly by their gender-role expectations for their offspring [62], as reflected by the fact that most single parents in the interviews gave their offspring the right to decide their affairs partially, such as choosing clothes or interest classes. Parents' gender role expectations, implicitly conveyed through various parent-child interactions in daily life, will eventually affect their offspring's gender role formation.

Single parents with more egalitarian gender attitudes' expectations of their offspring's gender roles are less influenced by traditional gender stereotypes; their children's gender role formation is affected by more open gender parenting attitudes and behaviours and daily parent-child interactions. During this process, other people, including grandparents, teachers, and peers, have a horizontal socialising influence on the children, modifying or reinforcing the single parents' gender parenting. The children's gender roles were ultimately determined under the combined influence of these multiple factors.

### 4.2 The discrepancy between attitude and behaviour of single parent's gender role education

In accordance with previous findings, our results further conformed that there is the discrepancy between the attitude and behaviour of single parents' gender role education [63]. Most single parents cognitively agreed that gender roles should be equalised and children should be free to develop in employment and other areas without traditional gender stereotypes. However, they still communicated their expectations authoritatively and used coercive “corrective” measures when their children's gendered behaviours exceeded certain limits. Chinese traditional gender stereotypes—e.g., that men are better suited to science and engineering and women to liberal arts—still strongly influence single parents' gender parenting, suggesting culture plays a powerful explanatory role in the persistent gender inequality in the division of labour [64].

This inconsistency between cognition and behaviour could be because single parents' attitudes toward gender equality are not strong enough. There is still a traditional gendered division of labour in social life; for example, nursing, domestic work, kindergarten teaching, and similar occupations are dominated by women, while men inevitably dominate engineering, machinery, manual labour, and similar professions. This gendered workplace impacts and limits single parents' concepts of gender equality, preventing them from being applied across the board. Studies have shown that while parents do not perceive gender differences in their parenting, the amount of time they spend with their children varies according to their children's physiological sex [65].

Previous studies have found that single parents' poor education efforts may be due to their lack of effective methods [66] and difficulty grasping the relationship between kindness and strictness [32]. Single parents must devote double the time and energy to raising their children alone because financial pressures are often heavier after a divorce, exposing them to longer working hours, reduced health, and lower quality of life [67]. The result is that they may not have the appropriate capital to expand their social resources. This, coupled with the psychological pain and loss caused by divorce, widowhood, etc., means they may be reluctant to participate in social activities. The combination of these disadvantageous effects narrows single parents’ cultural horizons and makes them more lacking in scientific approaches to parenting.

This suggests that the government and relevant departments should effectively help single-parent families increase their social resources and provide effective support policies to meet parents' and children's special needs. In addition, the single parents in this study tried to uphold gender equality and respect their children's free development in terms of cognition, but it was difficult to achieve unity in their cognition and behaviour. Therefore, single parents should strengthen their more equal gender concepts, continue to learn scientific and advanced gender parenting methods, and implement open parenting in their behaviour rather than just staying at the cognitive level.

### 4.3 The intergenerational transmission of gender roles consists explicit and implicit pathways

The intergenerational transmission of gender roles has two major pathways: explicit and implicit. This finding was consistent with previous studies [68,69]. The explicit path is dominated by parents' direct verbal instructions to their children, reflected in occupational expectations for their children and the choice of toys and clothing. Explicit parenting is vulnerable to conscious control [70], better reflecting single parents' tendency toward egalitarian gender perceptions. However, single parents have different behaviours when communicating with children of a different physiological sex. Single fathers were more passive and withdrawn in parent-child communications than single mothers, perhaps due to gender differences (e.g., “silent father's love” or “strict father and sensitive mother “) emphasised in Chinese traditional culture [71]. In contrast, research suggests that a high-quality parental presence facilitates children's gender role formation and development, while passive or poor communication is detrimental to children's formation of masculine gender identity. Therefore, fathers need to work harder than mothers to balance intimacy and separation from their offspring and be more flexible in responding to their adolescent children's different developmental goals [72].

The implicit path refers to single parents imperceptibly transmitting their gender role concepts through daily parent-child interactions, such as parent-child activities, housework, and tutoring. Parents' gender behaviour in daily life was more likely to be the object of imitation than their direct gender parenting. Some studies have found that parents' gender behaviour is a better predictor of offspring's gender role attitudes than their gender beliefs [73]. Therefore, single parents' long-term androgynous behaviour is conducive to forming androgynous gender role concepts in their offspring. In the interview, some offspring said they no longer agreed with traditional gender stereotypes because of their single father's feminine behaviours, such as washing clothes, cooking, and braiding hair.

Studies have confirmed the correlation between offspring's perceived parenting style and gender identity [74]. Single parents' gender differences in daily parent-child communication inevitably influence their offspring's perceptions of gender roles. Harmonious parent-child interactions are the first source of children's complete gender role development [75]. Single-parent children already face the absence of one parental gender; resentment towards their absent parents would be detrimental to children's development of androgynous gender roles [76]. Therefore, single parents should be proactive in their parent-child communications. Single fathers, especially, should break the shackles of “strict fatherhood” and have good and effective parent-child communications. In addition, single parents should make it clear that divorce is an issue between parents and avoid denying or deriding their ex-spouse in front of their children to avoid prejudicing them against a certain gender. In daily life, single parents' androgynous gender role behaviour can serve as a model of social learning, subtly influencing their children's gender role development. Therefore, single parents should not only voice gender equality concepts in their parent-child communications, they should also strive to adopt gender equality attitudes in their daily life. This can promote the formation of androgynous traits in their children.

Interventions targeting single parents should prioritize resources and support to improve their parenting skills and promote positive parent-child interactions. For example, workshops, counseling services, and support groups specifically for single parents could provide guidance for effective communication strategies, conflict resolution and promotion of gender equity in family dynamics. In addition, providing community resources such as child care services, educational programs, and mentoring opportunities could alleviate some of the single parents' life challenges and allow them to devote quality time to nurturing their children's development. In summary, interventions for single parents should emphasize effective parent-child communication, promote gender equity in daily life, and recognize the importance of positive and harmonious relationships in promoting the healthy development of children's gender roles.

### 4.4 Grandparental education and horizontal socialisation are important complementary forms

In line with previous works, our results also found that other family members (such as grandparents) in the home environment also influence children's gender roles. Teachers can reinforce or modify single-parent children's gender parenting, and peers are an important reference for children's gender role development [77,78]. This “non-parental influence” is called horizontal socialisation or oblique socialisation of children's gender role development [79].

The Chinese family model is characterized as “inhabited by small colonies” and strongly emphasised the value of the extended family. Due to China's economic and social development, parents must devote more time and energy to working, and “intergenerational education” has become an important supplementary form of parent-child education [80]. As such, it is common in Chinese families for grandparents to help care for their grandchildren. Regardless of whether the three generations live together, grandparents will help oversee children's daily lives by helping with laundry, cooking, and homework. Most interviewed families mentioned the influence of grandparental education on their children's gender roles, echoing other studies' findings on intergenerational differences in gender role stereotypes [81]. However, regardless of grandparents' openness or stereotypes, children in single-parent families are likely to be influenced by their grandparents' gender perceptions.

In addition, China has emphasised respect for teachers since ancient times, and parents in modern compulsory education often emphasize that children should ‘*listen to their teachers*’, which gives teachers an important role in children's gender role development. Some children mentioned that their teachers ‘*say the same thing*’ as their parents do in their gender parenting. Teachers' education can complement and modify parents' gender parenting through verbal and non-verbal interactions. This finding was consistent with previous study [82]: teachers' gender-differentiated attention, treatment, and guidance can influence students' eventual gender role formation. Children also take peer groups as an important reference object when forming their gender roles; as mentioned in the interview, ‘*all the students in our class are also* … ’ Campus peer groups have the effect of “abandoning the bad and promoting the good” in adolescents' personality development, supporting each other academically and emotionally and taunting children who play games that do not correspond to their physiological sex; children will intentionally adjust their behaviour to conform to gender role norms to be appreciated by their peers. Thus, school is an important personality socialisation site and plays an important role in guiding children's gender role development.

Vertical and horizontal socialisation mechanisms play interdependent roles in gender role formation [83]. Therefore, to help their grandchildren realize their gender role development, grandparents must be aware that they are not only “babysitters”, but also transmitters of gender knowledge and culture. They should consciously establish gender equality in their interactions with children. In addition, androgynous education is gradually being enlightened on modern Chinese campuses. Teachers must develop their awareness of gender equality so students can freely develop their interests and strengths in a gender-equal environment.

## 5 Conclusion

This study used semi-structured interviews to collect saturated data from 29 single-parent families (58 persons in total) on a household basis. It further constructed the mechanism of intergenerational transmission of gender roles in single-parent families through the qualitative research strategy of grounded theory. The mechanism presented four conclusions. Firstly, as the starting point of the intergenerational transmission of gender roles, the parents' gender role cognition influences their gender parenting attitudes and behaviours, which in turn influences the formation of the children's gender roles. Secondly, there is a discrepancy between the attitude and behaviour of single parents' gender role education. Although most parents in the interviews agreed that gender roles should be equalised in parenting attitudes, they were still prone to be constrained by traditional gender concepts in their behaviours, and took coercive measures to adjust their children's behaviours that were beyond their acceptance. Furthermore, the intergenerational transmission of gender roles consists of explicit and implicit pathways. The explicit pathway encompasses parents' direct verbal instructions to their children, while the implicit pathway involves single parents shaping their children's gender roles through various daily parent-child interactions, such as outdoor activities, household chores, and academic tutoring. Finally, the mechanism points out that grandparental education and horizontal socialisation are important complementary forms. The results of the study enriched the theoretical results of the intergenerational transmission of gender roles in single-parent families, and also had a guiding role in constructing the children's gender roles in practice.

## 6 Limitations and implications for future research

There are several limitations to the current study that should be acknowledged. First, it used a cross-sectional design with a family-based qualitative research approach to explore the mechanisms of intergenerational transmission of gender roles in single-parent families. Grounded theory requires in-depth interviews with respondents to uncover as much underlying information as possible [21]. To ensure smooth data collection, we chose secondary school students with more developed communication skills as interviewees. However, as some studies have indicated that children's gender roles develop at an earlier age [84], future research could use longitudinal studies to explore the mechanisms of gender role transmission within families at early childhood stages. Combining the breadth of quantitative research with the depth of qualitative research would help to validate and extend this study's findings.

Second, the sample of this study was limited to the Chinese single-parent families, therefore the data may not be replicable to other cultural backgrounds. Some family background characteristics were not included in the sampling structure, such as the time since the divorce, whether the ex-spouse and offspring were in contact, and whether the children lived with their grandparents after the divorce. Future research could be refined in these areas to explore the effects of different variables on the intergenerational transmission of gender roles in single-parent families.

Third, this study focused on how gender role perceptions were transmitted from single parents to their offspring. Although perceptions and attitudes were mainly transmitted from parents to children, the possibility that children may reciprocally influence their parents cannot be completely ruled out. Thus, the possible interaction between parental and offspring gender roles deserves attention in future studies.

## Data availability statement

Data will be made available on request.

## Ethics statement

All procedures performed in research involving human participants conform to the ethical standards of the Institutional Research Council and conform to the 1964 Helsinki Declaration and subsequent amendments. The study was approved by the Institutional Review Board (or Ethics Committee) of Soochow University (protocol code KY20220564B).

## Funding

The work was supported by the National Social Science Foundation of China (grant numbers 18BRK039). In addition, we sincerely thank the teachers who supported and assisted the project research and the participants who participated in the project.

## CRediT authorship contribution statement

**I-Jun Chen:** Writing - review & editing, Writing - original draft, Funding acquisition, Formal analysis, Data curation, Conceptualization. **Zisong Chen:** Writing - original draft, Resources, Project administration.

## Declaration of competing interest

The authors declare that they have no known competing financial interests or personal relationships that could have appeared to influence the work reported in this paper.
