# Supplementary material for: A study of the mechanism for intergenerational transmission of gender roles in single-parent families
Source: Heliyon. 2023 Nov 28;9(12):e22952. doi: 10.1016/j.heliyon.2023.e22952 (PMC10696254; doi:10.1016/j.heliyon.2023.e22952)
Supplement: Multimedia component 1 [file mmc1.docx]

As a qualitative study, we use semi-structured interviews with specific interview questions that differ for each respondent, but in general we will have the following interview questions as the main interview framework:

- How would you describe your gender role in your family? How do you think your gender role affects your daily life, such as your work, education, leisure, and relationships?

- How do you perceive your parent's gender role in your family? How do you think your parent's gender role influences your own gender role development?

- How do you communicate with your parent about gender-related issues, such as expectations, stereotypes, values, and norms? How do you feel about your parent's views on gender roles?

- How do you cope with the challenges or difficulties that arise from living in a single-parent family? How do you think these challenges or difficulties affect your gender role development?

- How do you compare your gender role with those of your peers who live in two-parent families? How do you think living in a single-parent family or a two-parent family makes a difference in terms of gender role development?
